# Suspicion and treatment of severe sepsis. An overview of the prehospital chain of care

**DOI:** 10.1186/1757-7241-20-42

**Published:** 2012-06-27

**Authors:** Johan Herlitz, Angela Bång, Birgitta Wireklint-Sundström, Christer Axelsson, Anders Bremer, Magnus Hagiwara, Anders Jonsson, Lars Lundberg, Björn-Ove Suserud, Lars Ljungström

**Affiliations:** 1 School of Health Sciences, Research Centre PreHospen, University of Borås, The Prehospital Research Centre of Western Sweden, SE 501 90, Borås, Sweden; 2 Department of Infectious Diseases, Skövde Central Hospital, Skövde, Sweden; 3 Sahlgrenska University Hospital, SE 413 45, Göteborg, Sweden

**Keywords:** Sepsis, Dispatch centre, Emergency medical service

## Abstract

**Background:**

Sepsis is a life-threatening condition where the risk of death has been reported to be even higher than that associated with the major complications of atherosclerosis, i.e. myocardial infarction and stroke. In all three conditions, early treatment could limit organ dysfunction and thereby improve the prognosis.

**Aim:**

To describe what has been published in the literature a/ with regard to the association between delay until start of treatment and outcome in sepsis with the emphasis on the pre-hospital phase and b/ to present published data and the opportunity to improve various links in the pre-hospital chain of care in sepsis.

**Methods:**

A literature search was performed on the PubMed, Embase (Ovid SP) and Cochrane Library databases.

**Results:**

In overall terms, we found a small number of articles (n = 12 of 1,162 unique hits) which addressed the prehospital phase. For each hour of delay until the start of antibiotics, the prognosis appeared to become worse. However, there was no evidence that prehospital treatment improved the prognosis.

Studies indicated that about half of the patients with severe sepsis used the emergency medical service (EMS) for transport to hospital. Patients who used the EMS experienced a shorter delay to treatment with antibiotics and the start of early goal-directed therapy (EGDT). Among EMS-transported patients, those in whom the EMS staff already suspected sepsis at the scene had a shorter delay to treatment with antibiotics and the start of EGDT.

There are insufficient data on other links in the prehospital chain of care, i.e. patients, bystanders and dispatchers.

**Conclusion:**

Severe sepsis is a life-threatening condition. Previous studies suggest that, with every hour of delay until the start of antibiotics, the prognosis deteriorates. About half of the patients use the EMS. We need to know more about the present situation with regard to the different links in the prehospital chain of care in sepsis.

## Background

There are a number of conditions in medicine, where every minute counts and the time from the onset of symptoms until the delivery of life-saving treatment is of ultimate importance. During the last few decades, this has resulted in improvements in the early chain of care in out-of-hospital cardiac arrest, presumed acute coronary syndrome (ACS) and stroke. This has resulted in a major improvement in prognosis, particularly with regard to ACS but also in out-of-hospital cardiac arrest [1].

Severe sepsis is now considered to be the most common cause of death in non-coronary intensive care units. Approximately 150,000 people die every year in Europe and > 200,000 die annually in the United States [2].

It appears that the delay from symptom onset until the delivery of treatment is important for outcome [3-7].

However, aspects of the very early chain of care, i.e. the prehospital chain of care, have not been evaluated extensively. With improvement in the very early chain of care in sepsis treatment could hopefully start even earlier.

As with cardiac arrest, ACS and stroke, the prehospital chain of care can be divided into four links or perspectives: 1/ the patients’ perspective; 2/ the bystanders’ perspective; 3/ the dispatchers’ perspective and 4/ the emergency medical service’s (EMS) perspective.

Our hypothesis is that in each of these four links there is room for improvement and that improvement might result in a shortening in delay to treatment in sepsis.

In each of these four links, a number of questions arise which focus on patterns of reaction, reasons for delay, the association between delay and outcome and opportunities for improvement.

Table 1 shows the most relevant questions that were addressed in the survey with regard to these four links.

**Table 1:** 

**Questions**	**Answers**	**References**
**General**		
· Is there an association between delay from symptom onset until start of treatment and outcome?	Yes, however data with regard to the value of pre hospital treatment with antibiotics are controversial	3, 4, 5, 6, 7, 23, 24, 25, 27, 28, 29, 30
**patient's perspective**		
· What is the patient decision time?	n/a	
· Can we define factors associated with prehospital delay?	n/a	
· Why do patients with sepsis wait before deciding to go to hospital?	n/a	
**bystander's perspective**		
· What are their thoughts and feelings?	n/a	
· Which action do they take and why?	n/a	
· Can we identify bystanders of high-risk patients?	n/a	
**dispatcher's perspective**		
· What signs and symptoms appear to the dispatchers by telephone in sepsis?	n/a	
· How often do the dispatchers suspect sepsis?	n/a	
· Can a decision support system improve the accuracy of their prioritization?	n/a	
**The EMS perspective**		
· How often do patients with sepsis use the EMS?	In 50 – 60%	32, 33, 34
· How often do the EMS staffs suspect sepsis?	In 20 – 50%	32, 36
· Is it possible to improve the EMS staffs’ accuracy in detecting sepsis?	(1) Limited knowledge among EMS staff	38
	(2) Biochemical markers might improve outcome	39

n/a = not available.

### Definition of sepsis [8]

#### Sepsis

The diagnostic criteria can be divided into five domains.

1 General criteria

These include a/ fever (core temperature > 38.3 C) or hypothermia (i.e. core temperature < 36 C); b/ heart rate > 90 beats/min or > 2 SD above the normal value for age; c/ tachypnea; d/ altered mental status; e/ significant oedema or positive fluid balance and f/ hyperglycaemia (plasma glucose > 7.7 mmol/l in the absence of diabetes).

2 Inflammatory variables

a/ leukocytosis; b/ leukopenia; c/ normal leucocyte count but > 10% immature forms; d/ plasma C-reactive protein > 2SD above normal limit; e/ plasma procalcitonin > 2 SD above normal limit.

3 Haemodynamic variables

a/ arterial hypotension (systolic blood pressure < 90 mmHg); b/ SVO_2_ > 70%; c/ cardiac index > 3.5 L/min

4 Organ dysfunction variables

a/ arterial hypoxemia; b/ acute oliguria; c/ creatinine increase; d/ coagulation abnormality; e/ ileus; f/ thrombocytopenia; g/ hyperbilirubinemia

5 Tissue perfusion variables

a/ hyperlactatemia; b/ reduced capillary filling.

It is important to stress that few if any patients in the early stages of the inflammatory responses to infection are diagnosed via four arbitrary criteria.

Instead, the health care provider, at bedside, identifies “myriad symptoms” and, regardless of evident infection, declares the patient to “look septic”.

### Severe sepsis

The definition of severe sepsis relates to sepsis complicated by organ dysfunction. Organ dysfunction can be defined using the definition formulated by Marshall et al. [9] or the definition used for the Sequential Organ Failure Assessment (SOFA) score [10] as suggested by ref [8] and [11].

#### Septic shock

This relates to the state of acute circulatory failure characterised by persistent arterial hypotension unexplained by other causes. Hypotension is defined as systolic blood pressure of < 90 mm Hg (or in children < 2SD below the norm for their age).

### Definition of delay

The delay which is mostly referred to in the acute phase of a life-threatening condition is the delay from the onset of symptoms until the start of treatment. When it comes to the treatment of sepsis, there are three major aspects of treatment, i.e. antibiotics, fluids and respiratory support. To date, these treatments have generally been started after admission to hospital [12-20].

One problem that might appear in sepsis is the definition of symptom onset. In all probability, many patients will have problems giving an exact time for the onset of symptoms, whereas others can describe the time of the symptom onset more exactly. This is a problem that is not unique to sepsis. Similar problems have been raised both in acute myocardial infarction [21] and in stroke [22].

When including the prehospital phase in the acute chain of care, the delay from symptom onset until the delivery of treatment can be divided into patient delay, i.e. the delay from the onset of symptoms until the patient calls for an ambulance or contacts other health-care providers, and system delay, i.e. the delay between the first contact with health-care providers and the start of treatment.

The aim of this survey was to describe what has been published in the literature with regard to a/ the association between delay until start of treatment and outcome in sepsis with the emphasis on the prehospital phase and b/ present knowledge and the opportunity to improve various links in the prehospital chain of care in sepsis. The ultimate goal is to find new ways to shorten delay to treatment in sepsis and thereby improve survival.

## Methods

In June 2011, literature searches were performed in the PubMed, EMBASE (Ovid SP) and Cochrane Library databases. Variations of the following terms were used, adapted for each database:

septicaemia, sepsis, patient delays, bystander effect, witness, dispatch, Emergency Medical Services, emergency health service, ambulances.

An example of the number of hits is shown below for Embase (Table 1).

**Figure 1:**
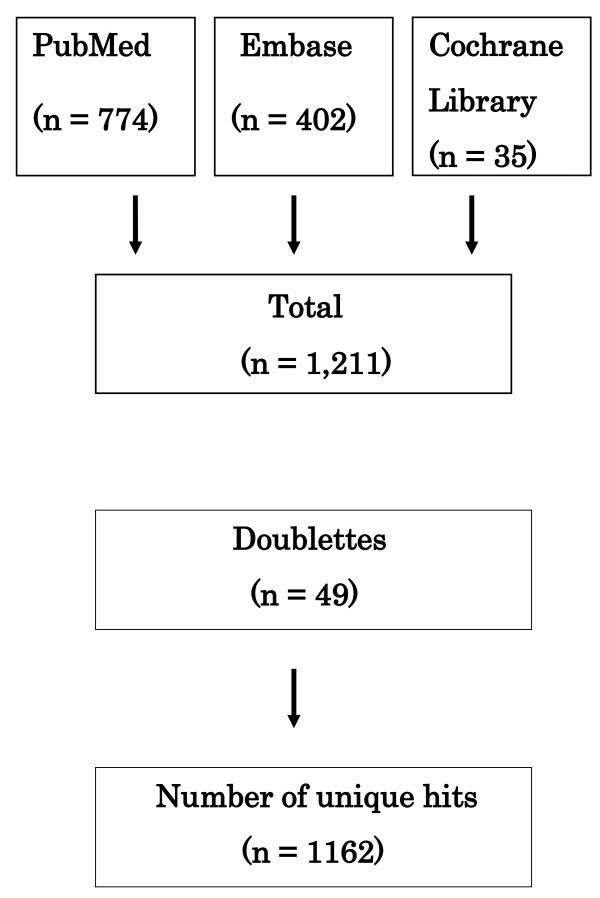
Number of hits in the various databases.

The number of hits fulfilling the criteria given is shown in Figure 1.

Table 3 shows the 12 articles which dealt with the prehospital setting and their major aims and conclusions.

**Table 2:** EMBASE 2011-07-06

Database(s): EMBASE 1980 to present		
Search strategy:		
**#**	**Searches**	**Results**
1	exp septicaemia/or septicaemia.mp.	18,156
2	sepsis.mp. or exp sepsis/	143,927
3	patient delays.mp.	63
4	exp bystander effect/or bystander.mp	5,711
5	exp witness/or witnessed.mp.	8,488
6	dispatch.mp	914
7	patient delays.af.	63
8	patient delay.af.	448
9	(witness or witnesses or witnessed).af	21,125
10	bystander.af.	5,711
11	dispatch.af.	928
12	(septicaemia or sepsis).af.	112,005
13	Emergency Medical Services.mp. or exp emergency health service/	51,496
14	Emergency Medical Services.af.	6,827
16	ambulances.mp. or exp ambulance/	6,776
16	(ambulance or ambulances).af.	10,308
17	1 or 2 or 12	147,364
18	3 or 4 or 5 or 6 or 7 or 8 or 9 or 10 or 11 or 13 or 14 or 15 or 16	85,897
19	17 and 18	609
20	limit 19 to Embase and (Danish or English or Norwegian or Swedish)	402

**Table 3:** Prehospital studies of sepsis

**Ref**	**Year**	**Aim**	**n**	**Results**
**A. Prehospital treatment**				
**(1) Meningococcal sepsis**				
23	1998	To assess the effect of antibiotics given by GP	32	Higher mortality among patients who received antibiotics
24	2002	To assess the effect of antibiotics given by GP	534	The effect of prehospital antibiotics appeared to be dependent on hospital care
25	2006	To explore mortality and morbidity after parenteral penicillin in children	158	Children who were given antibiotics had a more severe disease on admission to hospital
26	2005	Audit to determine the clinical appropriateness of administrations of benzyl penicillin by paramedics	69	Paramedic compliance with guidelines was low (78% failures)
**(2) Fluids**				
36	2010	To determine the delivery of out-of -hospital fluids in severe sepsis	52	Forty-eight per cent received intravenous fluids
**B. Impact of EMS on care of sepsis patients**				
32	2010	To evaluate early recognition and treatment in relation to EMS care	311	Patients who used the EMS had more organ failure but a shorter time to antibiotics and EGDT
33	2010	To characterise patients with sepsis in relation to the use of the EMS	4,613	EMS patients were more likely to present with severe sepsis
34	2010	To describe out-of-hospital characteristics and EMS care among patients with severe sepsis who used the EMS	216	Out-of-hospital variables were associated with organ dysfunction at the ED
42	2011	To assess the impact of the EMS on time to antibiotics, intravenous fluids and mortality in severe sepsis	963	Out-of-hospital care was associated with improved in-hospital processes but not mortality
**C. Prediction of outcome**				
39	2009	To consider how prehospital staff can improve the outcome in severe sepsis		The article suggests that antibiotics should be given in the prehospital setting and that lactate should be measured
35	2007	To assess the predictive effect of physiological elements commonly reported in the out-of-hospital setting on the outcome in severe sepsis	63	The out-of-hospital shock index and respiratory rate are highly predictive of ICU admission
**D. Knowledge and attitudes regarding sepsis among EMS staff**				
38	2010	To assess the knowledge and attitudes in the diagnosis and management of sepsis in the USA	226	Poor understanding of the principles of diagnosis and management of sepsis was observed

GP = General practitioner.

EMS = Emergency Medical Services.

EGDT = Early Goal Directed Therapy.

ED = Emergency Department.

## Results

### Association between delay to delivery of treatment and outcome (Table 1)

Some studies have reported an association between delay to the delivery of antibiotics and outcome in severe infection [6,7].

However, several studies have reported that children who received parenteral penicillin from general practitioners (GP) prior to arrival in hospital had a more severe outcome than those that did not [23-25].

One further study showed that paramedic compliance with guidelines when administering benzyl penicillin in presumed meningococcal disease in the prehospital setting was relatively low [26].

It was suggested that patients who received penicillin prior to arrival in hospital were more severely ill [23]. In the majority of these cases, the patients were suffering from meningococcal sepsis, which has been reported to have an extremely poor prognosis.

However, others have reported that the prehospital administration of antibiotics is associated with a more favourable outcome [27-30].

During the last few decades, “Early goal-directed therapy” (EGDT) in sepsis has been introduced at emergency departments (ED) in many countries. This treatment algorithm has been used in particular among patients with septic shock unresponsive to fluid challenges [3].

### The four links in the chain of care in the prehospital setting

#### The patient

##### *Decision time and overall prehospital delay*

The delay from the onset of symptoms to the decision to go to hospital or the decision to call for the EMS is insufficiently reported in the literature. Similarly, the overall prehospital delay from the onset of symptoms to arrival in hospital and factors associated with this delay are not known.

#### The bystander

In other conditions such as ACS and stroke, the bystander (often a wife or husband) is looked upon as a key link, since the patient is often suffering from denial and, in stroke in particular, is unable to think clearly [31]. Whether this is true also for sepsis is not well described in the literature.

#### The dispatch centre

Among patients with sepsis where the EMS has been called upon, the dispatch centre and the dispatchers play a key role.

The prioritisation and the dispatcher’s possible suspicion of a life-threatening condition might be of ultimate importance for the patient’s outcome. There is, unfortunately, no information in the literature with regard to these issues.

#### The EMS

##### *How often do patients with sepsis use the EMS?*

In one prospective observational study including ED patients with severe sepsis treated with EGDT, 51% were transported by the EMS [32]. Others report that the EMS provided care for about 60% of patients with severe sepsis [33,34].

##### *How often do the EMS staff suspect sepsis and, if so, are they able to predict outcome?*

In one report, the EMS staff had a primary impression of sepsis in 21% of EMS-transported sepsis patients [32].

In another report, it was shown that the out-of-hospital shock index and respiratory rate were highly predictive of intensive care unit admission [35]. In another study, only half of 52 patients with severe sepsis received out-of-hospital fluid [36].

##### *Which criteria do they use?*

In the prehospital setting, the health-care providers can obtain valuable information from general variables (hyper- or hypothermia, elevated heart rate and tachypnea).

They can also obtain valuable information from hemodynamic variables (hypotension), as well as signs of organ dysfunction (arterial hypoxemia).

As a result, the combination of elevated heart rate, an oxygen saturation of < 90% and a respiratory rate of > 30 per minutes, in combination with hyper- or hypothermia, should raise a strong suspicion of severe sepsis [37]. If this is combined with systolic blood pressure of below 90 mmHg, there is a strong suspicion of septic shock [37].

##### *Do the EMS staff follow these criteria?*

This is not well reported in the literature. A recent report suggests limited knowledge among EMS staff regarding various aspects of sepsis [38].

##### *Is it possible to improve the EMS staff’s accuracy in detecting sepsis?*

It is not unlikely that education and feedback to the EMS staff might improve their alertness to detect sepsis already in the prehospital setting. It has been suggested that a point-of-care analysis of lactate prior to arrival in hospital might improve the detection rate still further [39].

## Discussion

There is no clear evidence from the literature that, if possible, the treatment of sepsis should start in the prehospital setting. However, it appears that the time to the delivery of treatment is important for outcome and the earlier such treatment is started the better. In this aspect there are many similarities between sepsis and acute myocardial infarction. In the latter “time is saved myocardium” is known since many years.

One striking observation was the lack of reports from a prehospital perspective with regard to the patients’, bystanders’ and dispatchers’ perspective. We need further knowledge in order to see improvements in these links in the prehospital chain of care. It is not easy to calculate room for improvement if we do not have sufficient knowledge about the situation at present.

The observation of a limited knowledge regarding sepsis among EMS staff suggests that there is room for improvement in the capability to recognise sepsis among patients, bystanders and dispatchers, as well as EMS staff. The way educational efforts to achieve these goals should be structured remains to be determined.

It is also time to try to assess whether structural changes in the early chain of care in sepsis should be made and evaluated.

### Should sepsis patients who use the EMS be directly transported to a sepsis treatment ward, bypassing the ED?

Among patients with an ST-elevation myocardial infarction and stroke who are transported by the EMS, it has become a common procedure to bypass the ED and transport the patient directly to the cath lab and also in some hospitals to a stroke unit [40,41].

In patients with sepsis, this has not yet been evaluated. The situation in sepsis might be different, as organisations have been introduced at the ED in many countries to take immediate care of these patients, thereby making direct transport to an intensive care less meaningful.

### How should we optimise the communication between the EMS staff and the hospital regarding sepsis patients?

In the future, it is most likely that the opportunity to detect sepsis prior to arrival in hospital will improve. In order to improve the communication between the prehospital and the hospital team as in acute myocardial infarction and stroke [40,41] a sepsis coordinator within the hospital ED might be required. In all probability, a “hot line” could be set up between the EMS staff and the sepsis co-ordinator and this would most probably increase the preparedness of the “hospital staff” responsible for the initial care of sepsis patients and thereby shorten the delay to the start of life-saving treatment.

### Is there an association between EMS detection of sepsis and start of treatment?

In a previous report, it was found that, if there was a written impression of sepsis among EMS-transported patients at the scene, there was a shorter delay to the start of antibiotics and the start of EGDT [33].

### Is there an association between the use of the EMS and treatment and outcome in sepsis?

In two previous surveys, it was found that, in prospective observational studies among patients with severe sepsis, those who used the EMS had a shorter delay to the start of antibiotics and to the start of EGDT compared with those patients who did not use the EMS [32,42]. However, no previous study has confirmed an association between the use of EMS and outcome in sepsis.

### Biochemical detection of sepsis

Biochemical detection of various diseases including acute myocardial infarction in the prehospital setting is uncommon. In sepsis only lactate has been suggested as such a marker at present.

### How easy is it to tell when a common cold or flu turns into pneumonia and ultimately sepsis?

There is no distinct answer to this question. In the prehospital setting one might answer: “Follow your MEWS”, Modified Early Warning Signs. When the respiratory rate rises to nearly 30/min or the oxygen saturation deteriorates below 90% or systolic blood pressure decreases below 90 mmHg or the level of consciousness is decreasing, then one must suspect a bacterial complication.

## Conclusion

Severe sepsis is a life-threatening condition. Previous studies suggest that, with every hour of delay until the start of antibiotics, the prognosis deteriorates. About half of patients use the EMS and less than half of sepsis cases are detected by EMS staff. We need to know more about the present situation with regard to the different links in the prehospital chain of care in sepsis.

## Competing interests

The authors declare that they have no competing interests.

## Authors’ contributions

JH is responsible for the design of the manuscript, the literature search and the writing of the manuscript. AB has contributed constructive comments. BW has contributed constructive comments. CA has contributed constructive comments. AB has contributed constructive comments and references. MH has contributed constructive comments and references. AJ has contributed constructive comments. LL has contributed constructive comments. BS contributed constructive comments. LL has contributed valuable background information which was of importance for the design and content of the manuscript. All authors read and approved the final manuscript.
